# Punitive policing and associated substance use risks among HIV-positive people in Russia who inject drugs

**DOI:** 10.7448/IAS.17.1.19043

**Published:** 2014-07-09

**Authors:** Karsten Lunze, Anita Raj, Debbie M. Cheng, Emily K. Quinn, Carly Bridden, Elena Blokhina, Alexander Y. Walley, Evgeny Krupitsky, Jeffrey H. Samet

**Affiliations:** 1 Boston University School of Medicine, Boston, MA, USA; 2 Division of Global Public Health, University of California, San Diego, CA, USA; 3 Department of Biostatistics, Boston University School of Public Health, Boston, MA, USA; 4 Department of Medicine, Section of General Internal Medicine, Boston Medical Center, Boston, MA, USA; 5 Laboratory of Clinical Psychopharmacology of Addictions, St. Petersburg Pavlov State Medical University, St. Petersburg, Russia

**Keywords:** human rights, police involvement, PLHA, injection drug use, key populations, Russian Federation

## Abstract

**Introduction:**

Drug law enforcement is part of the HIV risk environment among people who inject drugs (PWID). Punitive policing practices such as extrajudicial arrests for needle possession and police planting of drugs have been described anecdotally in Russia, but these experiences and their associations with risky drug behaviours have not been quantified. This study aims to quantify the burden of extrajudicial police arrests among a cohort of HIV-positive PWID in Russia and to explore its links to drug-related health outcomes.

**Methods:**

In a cross-sectional study of 582 HIV-positive people with lifetime injection drug use (IDU) in St. Petersburg, Russia, we estimated the prevalence of self-reported extrajudicial police arrests. We used multiple logistic regression to evaluate associations between arrests and the following outcomes: overdose, recent IDU and receptive needle sharing.

**Findings:**

This cohort's mean age was 29.8 years, 60.8% were male; 75.3% reported non-fatal drug overdose, 50.3% recent IDU and 47.3% receptive needle sharing. Extrajudicial arrests were reported by more than half (60.5%, 95% confidence interval [CI]: 56.5–64.5) and were associated with higher odds of non-fatal drug overdose (AOR 1.52, 95% CI: 1.02–2.25) but not with recent IDU (AOR 1.17, arrests were associated with receptive needle sharing (AOR 1.84, 95% CI: 1.09–3.09).

**Conclusions:**

Extrajudicial police arrests were common among this cohort of Russian HIV-positive PWID and associated with non-fatal overdose and, among those with recent IDU, receptive needle sharing. As a part of the HIV risk environment of PWIDs, these practices might contribute to HIV transmission and overdose mortality. Further research is needed to relate these findings to the operational environment of law enforcement and to better understand how police interventions among PWIDs can improve the HIV risk environment.

## Introduction

The HIV epidemic in the Russian Federation (Russia) has dramatically expanded in the past 15 years and is bridging from high-risk groups to the general population [1]. Close to 1 million people (1.1% of adults) in Russia are estimated to be HIV positive [2], 665,000 of which are registered with the government [3]. Among newly diagnosed HIV infections, the proportion of women has increased to almost half; 40% of incident cases are due to injection drug use (IDU), while the proportion of heterosexual transmission has increased to 43% [4].

In parallel, IDU has been rising since the break-up of the Soviet Union and the subsequent political and socioeconomic turbulence [5]. The United Nations Office on Drugs and Crime estimates that more than 2 million Russians, or 2.3% of the adult population, use opioids [6]. In Russia, 14.4% of people who inject drugs (PWID) are HIV positive [7], a proportion that has reached 60–82% in some urban centres [8]. Prevention of HIV transmission among high-risk groups such as PWID is a key strategy to control the epidemic and avoid the spread from PWID to their sexual partners and to the general population. While prevention strategies aimed at individual behaviour are crucial, they also need to address the environmental determinants that contribute to risks on an individual and community level (micro risk environment such as law enforcement practices) or structural level (macro-level environment such as laws, policies or wider social perspectives) [9].

Russia's political and economic transitions and the Russian government's policy resistance to risk or harm reduction [10] have disrupted environmental risk reduction efforts on various levels, including addiction treatment, harm reduction programmes and drug law enforcement [11]. The current international standard treatment for opioid dependence, opioid agonist therapy, is prohibited by Russian law [12]. While syringe possession is not illegal in Russia, programmes providing clean syringes or other harm reduction services to reduce substance use-related risks operate against considerable political resistance and have never been scaled-up in spite of positive external evaluations [13].

In the face of the widespread and growing drug use epidemic in Russia, police, attempting to address the many challenges in drug law enforcement, have been reported to use punitive practices. In ethnographic research conducted in Russia, police persecution and discrimination has emerged as an important factor associated with risk behaviour and as a prominent barrier for PWID to access HIV care [14]. A qualitative study conducted in various parts of Russia (i.e. Moscow, Barnaul and Volgograd) described *extrajudicial arrests* as arbitrary arrests without legal justification, or following the planting of evidence to formally justify arrest or detainment [15]. In qualitative Russian studies on HIV and health risk, extrajudicial arrests (i.e. arrests in the absence of illegal activities) were cited by PWID and reported to produce fear and terror in their daily lives [10, 14].

Globally, drug law enforcement practices often constitute human rights violations, and related evidence from studies outside of Russia suggest that police practices such as extrajudicial arrests, planting of false evidence and extrajudicial syringe confiscations were associated with HIV and substance use risks [16]. In a study from Mexico, syringe possession arrests were associated with receptive needle sharing [17]. In a US study, drug-related police arrests were associated with increased mortality from overdose [18]. While these policing practices aim to reduce substance use, studies have not found an association between increased levels of police activities and improvements in drug use behaviour [15, 19].

There is a lack of studies from Russia quantifying the extent of extrajudicial arrests, experienced by PWID and investigating potential links of these police practices with HIV and substance use risks. This study therefore aimed to estimate the prevalence of this police involvement in a cohort of HIV-positive PWID, as well as to evaluate the association between these police practices and HIV-related drug risk behaviours. Given the existing evidence from other countries, we hypothesized that extrajudicial arrests for needle possession and planting of drugs by the police are associated with increased odds of receptive needle sharing and non-fatal overdose, and with increased odds of recent IDU.

## Methods

**Figure 1:**
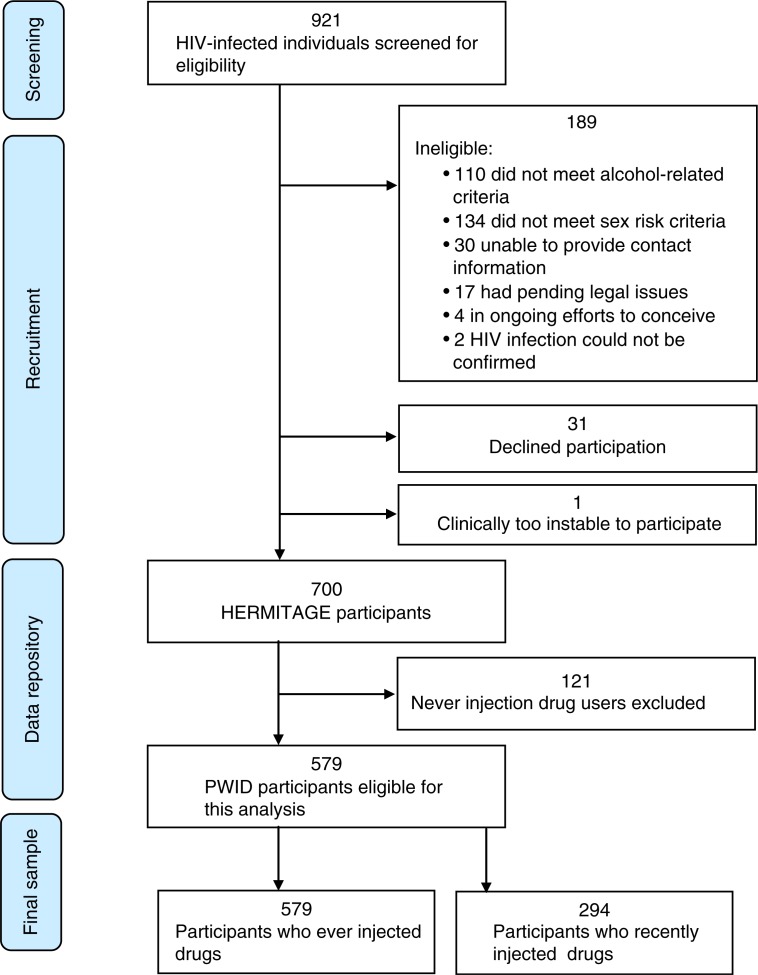
Inclusion of participants into HERMITAGE study and creation of data set for secondary analysis.

We carried out a baseline survey among the HERMITAGE (HIV Evolution in Russia – Mitigating Infection Transmission and Alcoholism in a Growing Epidemic) study, a randomized controlled trial of a behavioural intervention to reduce high-risk sexual activity and substance use. This trial is registered at ClinicalTrials.gov as NCT00483483. Participants were 700 HIV-positive heavy alcohol users with reported recent unprotected sex, who were recruited in St. Petersburg as previously described in detail [20]. We conducted a cross-sectional analysis of self-reported experiences with law enforcement officers of all 582 participants who reported ever having injected heroin (Figure 1). The Institutional Review Boards of Boston Medical Center and St Petersburg Pavlov State Medical University approved the study.

### Measures

Primary dependent variables were any non-fatal overdose and recent (i.e. past 30 day) IDU. Receptive needle sharing in the past 30 days (i.e. having used a potentially contaminated needle that someone else had used) was tested as a secondary dependent variable in a sub-analysis among respondents reporting recent IDU (*n*=294), as needle sharing applied only to that subgroup. The main independent variable was any self-reported lifetime extrajudicial arrest for needle possession or for needles or drugs planted by police. We used the term *extrajudicial arrests* because it included arrests for needle possession, which is not illegal in Russia, and the illegal planting of false evidence. Covariates included in the analyses were gender, educational status (primary school completion [grade 9] vs. higher), time since HIV diagnosis (under vs. over one year), sex trade involvement, any history of incarceration, heavy alcohol use in the past 30 days (i.e. any risky drinking as defined by the U.S. National Institute on Alcohol Abuse and Alcoholism, men: >4 drinks/day or >14 drinks/week; women: >3 drinks/day or >7 drinks/week), having ever been on antiretroviral treatment and frequency of drug injection.

### Analysis

We obtained descriptive statistics for all variables, stratified by the main independent variable, any extrajudicial arrest. We estimated proportions reporting extrajudicial arrests along with 95% confidence intervals. We used multiple logistic regression models to assess the association between extrajudicial arrests and each of the dichotomous outcomes (i.e. overdose, recent IDU and receptive needle sharing) controlling for potential confounders. To minimize the potential for collinearity, we verified that no pair of variables included in the regression models was highly correlated (i.e. *r*>0.40). We conducted all analyses with SAS software (version 9.2 SAS Institute, Cary, NC). We used two-sided tests and applied a significance level of 0.05.

## Results

As shown in Table 1, risk behaviours and adverse health outcomes were commonly reported. Most of the 582 PWID had experienced a drug overdose in their lifetime (75.3%). Almost half (47.3%) of the 294 recent drug users reported receptive needle sharing.

**Table 1:** Baseline demographic and clinical characteristics of a cohort of HIV-positive PWID in Russia (*N*=582) overall and stratified by reporting arrests or no arrests

	Overall, *n*=582	Arrests for needle possession or planted evidence, *n*=352	No arrests, *n*=230
Demographics			
Age mean (SD*)	29.8 (4.8)	30.1 (4.6)	29.2 (5.0)
Male	354 (60.8%)	234 (66.5%)	120 (52.2%)
Educational status beyond primary school	343 (58.9%)	207 (58.8%)	136 (59.1%)
Covariates			
Sex trade involvement	101 (17.4%)	67 (19.0%)	34 (14.8%)
Males only (*n*=354)	59 (16.7%)	44 (18.8%)	15 (12.5%)
Females only (*n*=228)	42 (18.4%)	23 (19.5%)	19 (17.3%)
Previous incarceration	249 (42.8%)	180 (51.1%)	69 (30.0%)
Males only (*n*=354)	184 (52.0%)	136 (58.1%)	48 (40.0%)
Females only (*n*=228)	65 (28.5%)	44 (27.3%)	21 (19.1%)
Ever been on ART	127 (21.8%)	78 (22.2%)	49 (21.3%)
Longer than 1 year since HIV diagnosis (*n*=581)	456 (78.5%)	278 (79.2%)	178 (77.4%)
Heavy alcohol use	472 (81.1%)	285 (81.0%)	187 (81.3%)
Any non-fatal overdose, lifetime	438 (75.3%)	276 (78.4%)	162 (70.4%)
Recent IDU (past 30 days)	294 (50.5%)	185 (52.6%)	109 (47.4%)
Number of injections (past 3 months), recent IDU; median (25th, 75th), *n*=294	50 (10, 81)	50 (12, 90)	40 (10, 60)
Receptive needle sharing (past 3 months, *n*=292)	138 (47.3%)	96 (52.5%)	42 (38.5%)
Hepatitis C antibody (*n*=514)	508 (98.8%)	306 (99.7%)	202 (97.6%)

SD, Standard Deviation.

Results in Table 2 show that the majority (60.5%, 95% CI: 56.5–64.5) of the 582 participants reported extrajudicial arrests (i.e. been arrested for carrying a syringe or after police planted syringes or drugs).

**Table 2:** Descriptive survey results on police involvement among HIV-positive PWID (*N*=582)

Police involvement	No.	Percentage (95% CI)
Had syringes taken by police	306	52.3 (48.5–56.6)
Been arrested for carrying a syringe	253	43.5 (39.4–47.5)
Been arrested after police planted syringes or drugs	259	44.5 (40.5–48.5)
Been arrested for carrying a syringe or after police planted syringes or drugs	352	60.5 (56.5–64.5)

As Table 3 shows, among the 582 study participants who had ever injected drugs, extrajudicial police arrests of PWID was significantly associated with drug overdose. We did not detect an association of extrajudicial police arrests with recent IDU among ever-drug users (i.e. we did not find police practices to be significantly associated with decreased odds of recent drug use. Among the 294 PWID reporting recent drug use, extrajudicial police arrests were associated with receptive needle sharing in the past three months (AOR 1.84, 95% CI: 1.09–3.09).

**Table 3:** Multivariable logistic regression models to evaluate associations between police arrests, and both overdose and IDU (*N*=582)

	Extrajudicial arrest (*N*=352)	Not arrested (*N*=230)		
			
Dependent variable	No. of events (%)	No. of events (%)	AOR* estimate (95% CI)	*p*
Drug overdose	276 (78.4)	162 (70.4)	1.52 (1.02, 2.25)	0.04
Recent IDU (past 30 days)	185 (52.6)	109 (47.4)	1.17 (0.82, 1.68)	0.38
Receptive needle sharing among PWID reporting recent IDU (past 30 days), N=292**	96 (52.5)	42 (38.5)	1.84 (1.09, 3.09)	0.02

* The model included the following covariates: age, gender, educational status, involvement in sex trade, history of incarceration, time since HIV diagnosis, past ART and heavy alcohol use. Analyses of receptive needle sharing were also adjusted for number of injections per 30 days

** among 292 current users: 183 arrested, 109 not arrested.

## Discussion

This study quantifies the extent of extrajudicial police involvement in a cohort of HIV-positive PWID in St Petersburg and examines its link with drug-related risk behaviours. This study documents that the majority of HIV-positive PWID in this Russian cohort experience extrajudicial arrests for needle or drug possession. Multivariable analyses show an association between these arrests and non-fatal overdose and, among those reporting recent IDU, receptive needle sharing.

These findings support the assertion that punitive drug law enforcement practices contribute to the HIV risk environment of Russian PWID. This is consistent with studies from other countries, where punitive policing practices have shown associations with risk behaviours and adverse health outcomes. In a study from Mexico, almost a third (32%) of PWID reported that police involvement led them to rush injections and share needles and syringes, and affected drug users’ decisions where to buy and use drugs because of the fear that police would interfere with their drug use [21]. Another study from Mexico among female sex workers found that HIV infection was independently associated with confiscation of syringes by police [22]. In a study from the USA, an increase in street police presence was found to be associated with a decrease in attendance at harm reduction programmes, particularly among minorities [23].

This study confirms an uneasy relationship between PWID and law enforcement officers. Law enforcement aims at a reduction of drug use, a goal that the public safety sector shares with the public health sector. However, these study results suggest that policing practices such as arrests for carrying needles, although not illegal in Russia, and planting drugs on PWID as a pretext for arrest and prosecution violates their rights and reinforces hazardous substance use behaviour. As a signatory to the Universal Declaration of Human Rights and other international human rights instruments, including the UN's International Covenant on Civil and Political Rights and the European Convention of Human Rights, Russia grants its citizens human rights as outlined in Chapter 2 of the Constitution adopted in 1993 [24]. Russia is also a member of the Council of Europe and as such bound to the decisions of the European Court of Human Rights with regards to its human rights obligations [25]. The violation of rights of PWID might be facilitated by the persistent high stigma in Russia and Eastern Europe against individuals with substance use and HIV infection [26], and by the power imbalance between police and PWID [27].

Thus, the law enforcement response to limit supply and use of drugs is part of a complex environment of exogenous risk factors impacting drug use risk. The police justify use of violence according to its own protocol when such acts enforce the legitimate goal of controlling the drug epidemic and reducing drug use. Among this cohort of HIV-positive PWID, however, punitive policing practices are associated with higher odds of risky behaviours. Although this study was not powered to detect a definitive reduction of drug use in relation to these police practices, its findings suggest that arrests are unlikely to have a substantive drug use deterrent effect. These findings are consistent with previous work suggesting that oppressive policing measures do little to deter drug use among PWID. In a population-level analysis in 89 US metropolitan areas, measures of legal repressiveness such as drug arrests and increasing police presence were not associated with drug use per capita, but were associated with higher HIV seroprevalence among PWID [28]. In Thailand, where drug policy is aggressively enforced, an increase in police presence was intended to deter drug use, but was not associated with a decrease in drug use [19]. Rather than having a beneficial effect on drug consumption, intensifying street policing reduced attendance at syringe exchange programmes [23].

This study quantifies the problem of extrajudicial police involvement in Russia and its association with substance use risks among HIV-positive PWID. Prior research on policing and health of PWID in Russia has been ethnographic and focused on perspectives of PWID on one side, and of police officers on the other. In a large qualitative study among more than 200 Russian PWID, informants identified fear of police interference at pharmacies and syringe exchange programmes as a primary factor limiting access to clean syringes. Respondents reported that fear of the police fed reluctance to carry used needles and syringes, and deterred them from safe disposal of needles and exchange for clean ones; fear of police also precluded their access to HIV and addiction care [29]. A qualitative exploration of police officers’ views on injecting drug use and needle and syringe access in Russia revealed that police officers were aware of drug users’ reluctance to carry injecting equipment linked to their fears of detention or arrest, but perceived PWID primarily as “potential criminals.” Police officers favoured a “pre-emptive” approach to the prevention of drug-related crime, such as the official registration of persons suspected or proven to be users of illicit drugs. Such registered persons are excluded from or limited in certain citizen rights such as parental rights (e.g. losing child custody or guardianship), driving licenses and certain professional occupations [30].

In order to reconcile this apparently adversarial relationship between PWID and police, we wish to redirect the policy discussion to the complementary public health and public safety goals of reducing drug use and related harms. Public health approaches seek to reduce the health and social consequences of substance use; public safety approaches seek to reduce drug-related crime and threats to public safety [31]. Both sectors could support each other's role. For example, public health professionals could communicate public safety messages, while public safety officers could function as first responders to overdoses or facilitate access for PWID to treatment and harm reduction programmes.

In concentrated epidemics, targeting the most-at-risk groups among whom HIV transmission occurs primarily is a key principle to prevent the spread of HIV among those groups, as well as preventing the bridging to the general population [32]. This study's findings emphasize the need for police and public health workers to collaborate in training and awareness to address the HIV risk environment of PWID. These partnerships should include PWID, health care providers, researchers, government officials and representatives from international organizations as well.

While our intention is for this study to inform law enforcement approaches from a health and HIV prevention point-of-view, we also recognize the need for public health planners and programmers to better understand police perspectives. Future research needs to not only gain an understanding of the operational environment of law enforcement in order to develop policing strategies compatible with public health goals, but also inform public health planners how to address police priorities in public health initiatives.

In the discussion of human rights of people living with HIV and of PWID, denial of harm reduction services, discriminatory access to HIV therapy, or coercive drug therapy are framed as health-related human rights abuses [33]. As this study confirms, human rights issues of PWID also include abusive policing practices, as they are part of their HIV risk environment. Given that some current policing practices in Russia reported in this study constitute human rights violations, and that these violations are associated with substance use hazards, protecting the human rights of PWID seems an essential part of improving their substance use and health behaviours. An outreach model that integrates legal support into psychosocial and medical prevention and treatment could facilitate rights-based harm reduction programming, for example. Such medico-legal partnerships and rights-based HIV and drug policies and programmes might not only protect the rights of PWID, but also contribute to preventing a further spread of HIV from PWID to Russia's general population. Transitioning policing strategies from the current punitive approaches to facilitating access for PWID to HIV and addiction services creates an opportunity for public health and public safety sectors to embrace each other's concepts in addressing the double epidemic of HIV and drug use in Russia.

This study has several limitations. Its observational and cross-sectional design precludes the ability to assign causality or ascertain the directionality of the observed association between police arrests and needle sharing and overdose. While police involvement might lead PWID to inject hazardously, causality might go in the reverse direction: those who engage in riskier injection behaviours and are at higher risk of drug overdose are more likely to come to police attention and therefore are more likely to be arrested. The wording of the study instrument and the observational, cross-sectional study design do not allow us to infer either causation or the temporal relationships of police involvement and associated outcomes. Measures of police involvement rely on self-report.

## Conclusions

This study suggests a link between extrajudicial arrests for needle or drug possession and adverse outcomes such as overdose and, among active PWIDs, receptive needle sharing. Mitigating the HIV epidemic in Russia will require not only prevention programmes to modify behaviours among most-at-risk populations on an individual level, but also to address policing practices as part of the HIV risk environment. This approach calls for human rights informed collaborations between police and public health to modify the risk environment of PWID in Russia.
